# Understanding factors affecting patient and public engagement and recruitment to digital health interventions: a systematic review of qualitative studies

**DOI:** 10.1186/s12911-016-0359-3

**Published:** 2016-09-15

**Authors:** Siobhan O’Connor, Peter Hanlon, Catherine A. O’Donnell, Sonia Garcia, Julie Glanville, Frances S. Mair

**Affiliations:** 1 General Practice and Primary Care, Institute of Health and Wellbeing, University of Glasgow, 1 Horslethill Rd, Glasgow, G12 9LX UK; 2 School of Nursing, Midwifery and Social Work, University of Manchester, Manchester, UK; 3 York Health Economics Consortium Ltd, York, UK

**Keywords:** Digital health, eHealth, Electronic health records, Telemedicine, Mobile applications, mHealth, Engagement, Recruitment, Barrier, Facilitator

## Abstract

**Background:**

Numerous types of digital health interventions (DHIs) are available to patients and the public but many factors affect their ability to engage and enrol in them. This systematic review aims to identify and synthesise the qualitative literature on barriers and facilitators to engagement and recruitment to DHIs to inform future implementation efforts.

**Methods:**

PubMed, MEDLINE, CINAHL, Embase, Scopus and the ACM Digital Library were searched for English language qualitative studies from 2000 – 2015 that discussed factors affecting engagement and enrolment in a range of DHIs (e.g. ‘telemedicine’, ‘mobile applications’, ‘personal health record’, ‘social networking’). Text mining and additional search strategies were used to identify 1,448 records. Two reviewers independently carried out paper screening, quality assessment, data extraction and analysis. Data was analysed using framework synthesis, informed by Normalization Process Theory, and Burden of Treatment Theory helped conceptualise the interpretation of results.

**Results:**

Nineteen publications were included in the review. Four overarching themes that affect patient and public engagement and enrolment in DHIs emerged; 1) personal agency and motivation; 2) personal life and values; 3) the engagement and recruitment approach; and 4) the quality of the DHI. The review also summarises engagement and recruitment strategies used. A preliminary **DI**gital Health **E**n**G**agement MOdel (DIEGO) was developed to highlight the key processes involved. Existing knowledge gaps are identified and a number of recommendations made for future research. Study limitations include English language publications and exclusion of grey literature.

**Conclusion:**

This review summarises and highlights the complexity of digital health engagement and recruitment processes and outlines issues that need to be addressed before patients and the public commit to digital health and it can be implemented effectively. More work is needed to create successful engagement strategies and better quality digital solutions that are personalised where possible and to gain clinical accreditation and endorsement when appropriate. More investment is also needed to improve computer literacy and ensure technologies are accessible and affordable for those who wish to sign up to them.

**Systematic review registration:**

International Prospective Register of Systematic Reviews CRD42015029846

**Electronic supplementary material:**

The online version of this article (doi:10.1186/s12911-016-0359-3) contains supplementary material, which is available to authorized users.

## Background

Patients are beginning to use a range of digital health interventions (DHIs) to manage chronic illness at home and support independent living and self-care, while remaining connected to health and care providers [1]. DHIs may address many of the problems patients experience with today’s health systems, such as poor access, uncoordinated care and increasingly costly healthcare [2]. Furthermore, DHIs aimed at the public are seen as one way to promote preventative health, potentially reducing health service utilisation and cost long-term [3]. DHIs range from telehealth and telecare systems [4], to patient portals and personal health records (PHRs) [5, 6], mobile health applications [7], and other online platforms and devices [8]. As the technology diversifies, miniaturises and becomes more interconnected, the shift towards using such DHIs will continue to grow.

However, numerous barriers prevent people from participating in evaluations of DHIs such as being too busy, feeling incapable of using the technology or disliking its’ impersonal nature [9, 10]. There are also factors that help patients and the public to engage with these electronic platforms such as personal motivation to improve health and learn new ways to manage illness [11]. Much of this evidence has been generated through quantitative methods, in particular Randomized Controlled Trials (RCTs), which provide little detail or context of the real-world difficulties individuals’ face [12, 13] such as the cost of the technology and issues around privacy and security [14]. Understanding these problems is particularly important as we move from recruiting to RCTs, to engaging and enrolling patients and the public in large-scale deployments of digital health in real world settings. This gap in knowledge is often referred to as the second translation gap, moving from initial concept testing and RCTs to full-scale implementation [15, 16].

Although an increasing number of qualitative studies have examined some of these issues, quite often they have focused on a particular patient population and a single piece of technology [17, 18]. Therefore, the literature is fragmented and does not present a clear picture of the barriers and facilitators people face when engaging and enrolling in all types of DHIs. Qualitative syntheses can aid our understanding of how complex interventions are embedded into daily routine, which can help to inform health policy and clinical practice [19, 20]. A qualitative review of public engagement with eHealth has been conducted [21] but the majority of included studies looked at people who searched for health information online only, so it is limited in terms of its technological scope and it was undertaken in 2009, six years ago, which is a long time in a fast moving area. The review also lacked any assessment of the quality of included studies and had no theoretical basis, thereby diminishing the lessons that can be drawn from it.

This paper aims to address the fragmentation of research evidence by systematically reviewing and synthesising the qualitative literature on barriers and facilitators patients’ and the public experience during engagement and recruitment to DHIs. It will also outline the strategies described to get people engaged and signed up to DHIs in the published literature. To address the lack of theoretical insights in this area, two empirically grounded theories will be utilised to aid in the conceptualisation of the complexities involved and develop a model of these processes. A series of recommendations about how patients and the public can be better supported to take up digital health products and services will also be outlined to improve the initial phases of the digital health implementation journey. Any outstanding research gaps will also be highlighted.

## Methods

A protocol was created and the review registered on PROSPERO, the International Register of Systematic Reviews (CRD42015029846, http://www.crd.york.ac.uk/PROSPERO/display_record.asp?ID=CRD42015029846).

### Search strategy

A scoping search was conducted to identify key papers and search terms to inform the design of the search protocol. This included three groups of concepts: (1) engagement and recruitment, (2) DHIs, and (3) barriers and facilitators. As it was thought important to capture the views of multiple stakeholders who would be aware of the experiences of patients and the public the population was not specified. A combination of MeSH headings, free text search terms and a novel text mining approach were used to narrow the considerable digital health literature and overcome the challenges of identifying relevant papers, which is described in detail elsewhere [22]. Six online bibliographic databases; CINAHL (EBSCHOHost), PubMed, Medline, Embase, Scopus and the ACM Digital Library, were searched for English language publications between January 1, 2000 and August 19, 2015 (see Additional file 1). Reference and citation tracking, the ‘Similar articles’ function in PubMed, personal knowledge, and contacting experts in the field were also utilised to identify relevant papers. Endnote was used to remove duplicate citations before screening.

### Selection criteria

Qualitative studies that explored the reasons why patients’ or the public engaged and enrolled in a range of digital health interventions were included (see Table 1).

**Table 1:** Inclusion and exclusion criteria used for the screening process

Inclusion criteria	
Study type	Publication date from 2000 present.
	Studies from any geographical location.
	English language.
	Original qualitative studies, studies involving secondary analysis of qualitative data or qualitative studies that are part of a mixed methods study (e.g. the study also has a quantitative component but the major component is qualitative and a qualitative methodology is described). The study must have direct contact with individuals or direct observation using any form of qualitative method.
Participant Type	Any individual (adult or child). This includes patients, the public and health professionals who would be aware of the experiences of these groups.
Type of digital health intervention	Any health intervention delivered by a digital technology (hypothetical or in development, simulated or real-world) which takes information from patients or the public or provides some form of advice or feedback about their health. This includes, but is not limited to: • Web-based interventions on personal computers (PCs) or mobile platforms, • Mobile health applications or apps, • Patient portals or personal health records, • Interventions delivered by short message service (SMS) or interactive voice recognition (IVR).
Setting	Any ‘usual’ setting (hypothetical or in development, simulated or real-world) such as primary, secondary or tertiary care, the home or workplace.
Phase of implementation	Engagement and recruitment phase of a digital health intervention, which can span from gauging an individual’s readiness for a digital health intervention, to the initial marketing or reach of the initiative, to actively signing individuals up to use the technology so they are registered on the digital application or system.
Exclusion criteria	
Study Type	Published pre 2000.
	Non English language.
	Grey literature/not published in a peer reviewed journal.
	Dissertation/thesis.
	Published abstracts or conference proceedings.
	Studies using the following methodologies: descriptive case studies, lexical studies that analyse natural language data presented as qualitative results; qualitative studies using questionnaires or other methods that do not involve direct contact or observation of participants.
	Any type of literature review, systematic review and meta-analyses, or a qualitative study that did not involve direct contact or observation of participants.
	Randomized Controlled Trials due to the large volume of literature on the difficulties recruiting to clinical trials that already exists [94].
	Commentary articles, written to convey opinion or stimulate research/discussion, with no research component.
Type of digital health intervention	Primary digital intervention is; telephone based with no additional technological function (e.g. telephone counselling or triaging service); Internet based with no additional interactive function (e.g. searching for health information online); or an implantable device that is remotely monitored
Setting	Any non-usual setting e.g. prison, armed forces in active duty.
Stage of implementation	Pre-implementation work based solely around designing the interface and functionality of the digital health intervention.
	The post engagement/recruitment phase will not be explored. For example: • why patients or the public use or do not use digital health interventions, • why they drop out (attrition) or fail to continue using them (retention), • their attitudes or beliefs towards digital health interventions, or their satisfaction with them outside of that pertaining directly to engagement and recruitment.

### Screening, data extraction and quality appraisal

The titles, abstracts and full papers were screened independently by two reviewers using DistillerSR software. Any discrepancies were discussed and disagreements adjudicated by a third party. A standardised data extraction template was then used which addressed a number of study characteristics (see Additional file 2). Text pertaining to barriers, facilitators, engagement and recruitment strategies, which included findings and interpretations written by the authors or participant quotes, were regarded as data and extracted for coding. Two reviewers independently performed a quality assessment using the 32-item Consolidated Criteria for Reporting Qualitative Research (COREQ) checklist [23, 24]. Although some would argue against such critical appraisal due to the unique philosophical and methodological underpinnings of qualitative work and the sometimes prescriptive use of such checklists [25, 26], others believe applying quality standards enables a more thorough exploration of the contribution of each study thereby improving the credibility of qualitative synthesis [27]. All articles meeting the inclusion criteria were retained, regardless of their quality, as even methodologically weak studies can sometimes offer valuable insights [28, 29].

### Data analysis

Our qualitative synthesis was informed by the framework approach [30, 31] as it provides a robust process to support analysis [32]. An empirically grounded theory, Normalization Process Theory (NPT) [33, 34], was used to underpin the process. NPT is a useful heuristic device to explain how people individually and collectively embed new interventions in everyday routine through four generative mechanisms: sense-making work; relational work; operational work; and appraisal work (see Table 2) and it has been used successfully in other systematic reviews [35, 36]. This provided a solid theoretical basis to develop a new conceptual model of digital health engagement and recruitment processes. Each item of extracted data was coded independently by two researchers. Coding clinics were held with a third researcher to ensure consistency of approach. Codes were compared and contrasted in a framework, then categorised and classified into higher order themes that were mapped onto the generative mechanisms of NPT. NVivo QSR 10.0 was used to facilitate analysis. During this process common themes began to emerge, indicating data saturation. Any negative data was carefully noted to ensure the new conceptual model was appropriate and any variances accounted for [37]. Burden of Treatment Theory (BOTT) was then used as a lens to develop recommendations for successfully enabling patients and the public to engage with digital health, as it describes how people cope with new interventions and enact self-care practices through their relationships with formal and informal health and care networks [38, 39].

**Table 2:** NPT Coding Framework

Coherence (CO)	Cognitive Participation (CP)	Collective Action (CA)	Reflexive Monitoring (RM)
*The* *sense-making* *work that people do individually and collectively when faced with engaging and enrolling in a digital health intervention*	*The* *relational work* *that people do individually and collectively to build and sustain engagement and enrolment in a digital health intervention*	*The* *operational work* *that people do by investing effort and resources to engage with and sign up to a digital health intervention*	*The* *appraisal work* *that people do to evaluate engagement and recruitment to a digital health intervention that affects them and others around them*
Differentiation (CO-d)	Enrolment (CP-e)	Skillset Workability (CA-sw)	Reconfiguration (RM-r)
Defining, dividing up and categorizing tasks	Recruiting the self and others to tasks	Allocating tasks and performances	Modifying or changing tasks
Communal Specification CO-cs)	Activation (CP-a)	Contextual Integration (CA-ci)	Communal Appraisal (RM-ca)
Making sense of shared versions of tasks	Organising a shared contribution to tasks	Supporting, resources and integrating tasks in their social contexts	Shared evaluation of contributions to tasks
Individual Specification (CO-is)	Initiation (CP-i)	Interactional Workability (CA-iw)	Individual Appraisal (RM-ia)
Making sense of personal versions of tasks	Organizing an individual contribution to tasks	Doing tasks, and achieving outcomes in practice	Individual evaluation of contributions to tasks
Internalization (CO-i)	Legitimation (CP-l)	Relational Integration (CA-ri)	Systematization (RM-s)
Learning how to do tasks in context	Making tasks the right thing to do	Developing confidence and communicating reliable knowledge about tasks	Organizing a reliable stock of knowledge about tasks

## Results

**Fig. 1:**
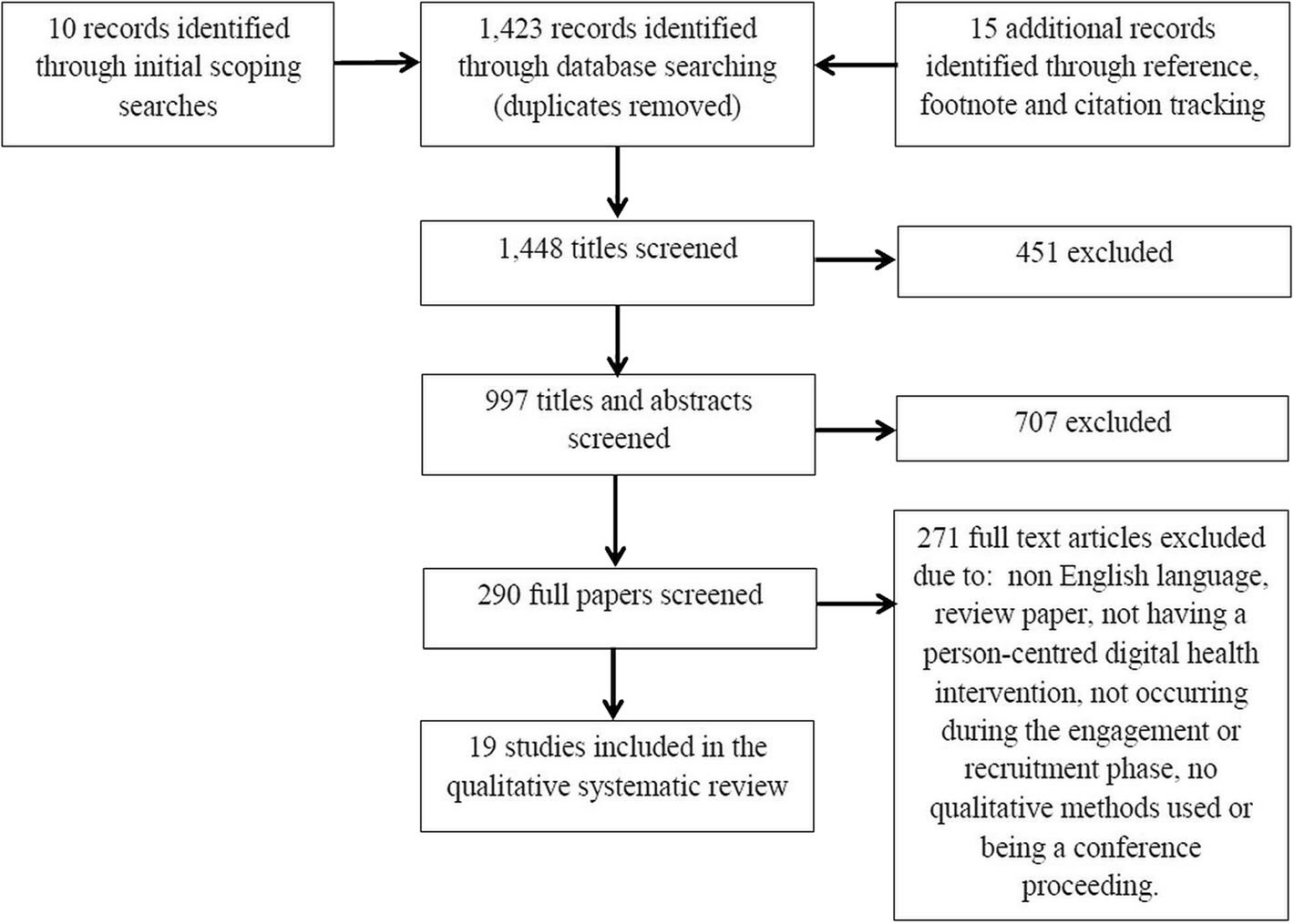
Preferred Reporting Items for Systematic Reviews and Meta-Analysis (PRISMA) flow diagram of search strategy to identify articles

In total, 1,448 records were identified, of which 290 full text articles were screened and 19 were selected for inclusion in the review (see Fig. 1). The reporting of this review follows the Enhancing Transparency in Reporting the Synthesis of Qualitative Research (ENTREQ) statement [40].

### Characteristics of included studies

A summary of the characteristics of included studies and participants can be found in Additional file 3. The included studies were published between 2005 and 2015, with the majority being published in the last four years. The studies were published in a number of countries with eight taking place in the United Kingdom [41–48], five in the United States [49–53], four in Canada [54–57] and one each in Norway [58] and Spain [59]. They spanned numerous types of DHIs including patient accessible electronic health records and PHRs [47, 48, 57], a telehealth system for diabetics [49], web-based sexual health and cognitive behavioural therapy services [42–45, 55, 56], an online appointment booking and patient provider communication system [46, 58], an Internet support group [53]; a social networking application [50]; and email, SMS or mobile phone based health promotion, smoking cessation or weight loss programmes [41, 51, 52, 59]. Only one study was a mixed intervention combining a pedometer with nutritional education and meal preparation training [54]. Fifteen studies were purely qualitative using a combination of interviews, focus groups, participant observation and documentary evidence [41–45, 47, 49, 52–59] with only four studies adopting mixed method approaches [46, 48, 50, 51]. The participants in the studies were patients, carers and healthy individuals from a variety of ages, genders, socioeconomic groups and ethnicities [42, 44, 45, 47, 48, 50–59] or were health professionals such as nurses or family doctors [43, 44, 46, 48, 49, 59]. Three studies had a mixture of other participants such as employees of large public and private companies, general practice staff and a range of individuals from local and national organisations affiliated with the implementation of a DHI [41, 46, 48]. However, several studies did not describe participant characteristics in detail: with three not depicting gender [44, 48, 49], four not portraying age [43, 44, 48, 49], nine not describing socio-economic status [43–46, 48, 49, 56, 57, 59], and eleven not highlighting ethnicity in detail [41, 43–46, 48, 49, 55, 57–59]. In general there was a trend towards younger and more middle aged people, rather than older adults, and those of “white” ethnicity.

#### Engagement and recruitment strategies

A range of engagement and recruitment strategies for DHIs were described. We classified engagement as any process by which patients’ and the public became aware of or understood a DHI for example through promotional efforts and marketing campaigns. These ranged from multiple forms of advertising to the use of health professionals, family and friends. DHIs were advertised on radio [47, 48], in print media such as newspapers; personal letters; posters on notice boards; printed flyers and leaflets [41, 46–49, 51], via electronic media e.g. television screens and digital notice boards and online via email, social media, website and Internet communities or forums [41, 46, 48]. More traditional forms of direct engagement were also employed such as consultations with health professionals [45, 47–49], employers [41], personal recommendations from family or friends [54] or being spoken to by research or management staff [46, 58]. Co-design activities were also utilised to get patients and the public involved in creating a DHI [42, 52, 55, 59]. We distinguished enrolment as any approach that involved people actively registering for or signing up to a DHI. Enrolment strategies were similarly wide ranging, with different levels of participation required from individuals. They included filling out paper based registration forms [45, 48, 58], sending a SMS text message [51], creating an online account or profile [41, 48, 51] or getting personal assistance from a health professional, administrator or researcher to do so [48, 49, 51], or in one particular instance consent was implied and an online account was automatically created [47]. In general, the engagement and enrolment strategies used in the included studies were not described in detail but are summarised in Table 3. There was insufficient data in the included studies to allow us to build a full taxonomy of engagement and enrolment strategies.

**Table 3:** List of digital health engagement and recruitment strategies

Engagement Strategy	
Advertising (Indirect)	Electronic media - television screens and digital notice boards Online media – email; social media; websites; Internet communities or forums Print media - newspaper advertising; personal letters; posters on notice boards; printed flyers and leaflets Radio
Personal Contact (Direct)	During a consultation with a health professional Research or management staff within a healthcare facility During a consultation with an employer Family, friends or peers Co-design activities
Recruitment Strategy	
Automatic	Consent is assumed and a digital profile or account is created
Electronic	Register online via a website
Paper based	Complete a paper based registration form
Personal Assistance	Healthcare professional helps to create a digital profile or account
Telephone or mobile phone	Telephone registration line Send a SMS text message

### Quality appraisal

The quality of reporting in the included studies varied with between 10 and 24 of the 32 items from the COREQ checklist (see Additional file 4) [23]. All 19 studies included the sample size, presented the main themes clearly and demonstrated consistency between the data collected and the findings. Seventeen provided some type of interview guide and described how participants were approached. Only one study reported repeating interviews and one returning transcripts to respondents. Overall the studies were of reasonable quality.

### Issues affecting digital health engagement and recruitment

Four major themes and several subthemes related to barriers and facilitators to engagement and recruitment in DHIs emerged (see Table 4). The four main themes are: 1) personal agency and motivation; 2) personal life and values; 3) engagement and recruitment approach; and 4) the quality of the DHI. Participant quotes are provided in the text to substantiate the data in each theme and more are available in Additional file 5.

**Table 4:** Factors affecting digital health engagement and recruitment

Barriers		Facilitators	
Themes 1: Personal Agency and Motivation			
Barrier Subtheme 1.1: Lack of Motivation	Lack of motivation to understand or improve health	Facilitator Subtheme 1.1: Personal Motivation	Motivation to understand and improve health
Barrier Subtheme 1.2: Awareness and understanding	Unaware of or lacks understanding of how a DHI could be helpful	Facilitator Subtheme 1.2: Awareness and understanding	Ability to understand a DHI and personal health data
Barrier Subtheme 1.3: Personal Agency (choice and control)	Alternative ways of documenting health information and managing illness	Facilitator Subtheme 1.3: Personal Agency (choice and control)	Ability to choose time and location of interaction with a DHI Ability to control electronic personal health data
Themes 2: Personal Life and Values			
Barrier Subtheme 2.1: Personal lifestyle	Busy lifestyle with competing priorities	Facilitator Subtheme 2.1: Personal lifestyle	DHI fits with personal lifestyle
Barrier Subtheme 2.2: Skills and equipment	Poor digital literacy	Facilitator Subtheme 2.2: Skills and equipment	Good digital literacy
	Lack of access to equipment and the Internet		Has or can afford computer equipment or mobile device, network connectivity and a data plan
	Cost of a DHI		
Barrier Subtheme 2.3: Privacy and security	Concern over the security and privacy of DHI information or interaction	Facilitator Subtheme 2.3: Privacy and security	Values the privacy and anonymity of DHI information or interaction
Theme 3: Engagement and Recruitment Approach			
Barrier Subtheme 3.1: Recruitment strategy	Difficulty understanding the recruitment message	Facilitator Subtheme 3.1: Recruitment strategy	Active promotion and engagement strategies
			Health professional acts as a gatekeeper
Barrier Subtheme 3.2: Direct support	Lack of support from family members, friends or peers	Facilitator Subtheme 3.2: Direct support	Support from family members, friends or peers offline
Barrier Subtheme 3.3: Personal advice	Lack of advice and recommendations from trusted sources	Facilitator Subtheme 3.3: Personal advice	Recommended by family members, friends or peers
Barrier Subtheme 3.4: Clinical endorsement	Lack of clinical endorsement and support for a DHI	Facilitator Subtheme 3.4: Clinical endorsement	Clinical accreditation and support for a DHI
Theme 4: Quality of the Digital Health Intervention			
Barrier Subtheme 4.1 and 4.2: Negative digital health experience (quality of information or interaction)	Impersonal DHI (poor quality information or interaction)	Facilitator Subtheme 4.1 and 4.2: Positive digital health experience (quality of information or interaction)	Open, honest digital interaction with healthcare provider
	Lack of trust in DHI information or interaction		Previous negative experience of health services without a DHI
	Digital health interaction could be abusive		Social support from peers online
Barrier Subtheme 4.3: Usability of the DHI	DHI is difficult to use	Facilitator Subtheme 4.3: Usability of the DHI	DHI is easy to enrol in and use (automated and integrated)
	Complex registration process		

#### Personal agency and motivation

The first theme that emerged concerned personal agency and motivation, as patients and members of the public who wanted to be healthy or have more choice and control over managing their wellbeing tended to engage and enrol in DHIs. They saw technology as a good way to maintain motivation to be physically active and lose weight, while preventing the onset of disease [41, 49, 54, 59]. Many people signed up to a DHI as it gave them the choice to access health information when and where it suited, which in some cases helped reduce anxiety [41, 43, 55, 56, 59]. People also liked the level of control technology offered in terms of monitoring and understanding health related behaviours, such as diet and exercise, or self-managing chronic conditions and this encouraged registration [48, 49, 57].

“*[I subscribed] to get the reminders, because if you’re sat, if you are in a lunch break and you’re sat at your desk just on the Internet and you’re not moving and you’re eating something that’s not good and then you get a reminder and it’s just: ‘have a walk!’, or something. Straight away there is a trigger in your mind and you think: ‘yeah, that’s right, I can do that!”* – Facilitator (CO-i) [41]

In contrast, a barrier to engaging for some was poor awareness of technology or seeing no value in the DHI offered or lacking the motivation to understand and improve their health through electronic data; this was often seen to be the role of their healthcare provider [48, 49]. For others the DHI was considered as a constant reminder of their failure to meet healthy goals and was thought to be discouraging [52, 54]. Technology was also viewed as potentially disruptive by some or purely for entertainment purposes by others and not for healthcare needs [47, 48, 52]. Many people already used other ways to manage their health or illness, such as recording data via paper based systems, gaining support through family, friends and health professionals, or maintaining physical activity levels. They preferred to continue using these alternative approaches than convert to electronic solutions [41, 46–48, 53, 55].

*“For me, it does not change anything because I am always in a car. I walk very little so I will feel even guilty for not having walked. I will look down at the low numbers and I’ll feel anxious.”* – Barrier (CO-is) [54]

#### Personal life and values

The second theme to emerge was how a busy personal life, with lots of competing priorities, affected patients and the public’s ability to engage with and enrol in DHIs. Those who thought the technology was relevant or could be tailored to their needs and it fitted easily into their personal life tended to sign up for it [41, 43, 52, 55–57, 59]. In addition, those who had or were already familiar with using technology [43, 49] and were digitally literate [43, 49, 57] found it easier to enroll as they had the right knowledge and skills to do so. Some people signed up as they liked the anonymity that online health services provided, feeling secure and free from the embarrassment and discrimination that they sometimes experienced in the real-world [43, 45, 47, 53, 55–57].

*“This is definitely a service I would use, not only for the convenience factor but I mean, no matter how old we are, it’s still an embarrassing issue for a lot of people.”* – Facilitator (CA-iw) [55]

Alternatively, where people had demanding careers, families with caring responsibilities or other pressures, it meant they had little time or enthusiasm for engaging with DHIs [41, 46–48, 50, 53, 54]. People were also worried about the privacy and security of personal health information as it could be compromised online and potentially disclosed to a partner, family, friend, co-worker, or employer or used by private industry or governments to infringe on their rights [42–44, 50, 52, 55, 56]. Digital literacy was another commonly cited barrier that hindered engagement as those who had little or no experience of using computer or mobile devices and lacked the necessary technical skills struggled to take part. In a few cases individuals had problems with English literacy as it was not their first language [44–49, 52, 55]. A lack of computer or mobile equipment and access to the Internet was another reason some people could not register for a DHI [44, 46–50]. For some this was due to the prohibitive costs involved and people’s inability to access affordable technologies [44, 50–52].

*“I’m very wary of the internet, we leave digital footprints wherever we go and you never know what’s going to come back and haunt you and I think the more that you are in a professional working environment the more you need to be careful about what you put online. You’ve got to keep it within certain parameters.”* – Barrier (CA-ri) [44]

#### Engagement and recruitment approach

The type of strategy or approach used to sign patients and the public up to DHIs was the third major theme that affected enrolment. Personal recommendations from trusted people such as family members, friends or peers was important and the support these social groups provided offline helped people to engage with and register for a technology, whereas those who lacked support often failed to sign up [41, 48, 50, 53, 54]. Active promotion and recruitment strategies, that were personalised where possible, were also beneficial as they helped reach the right audience and convince them to take part [41, 42, 46]. In one study a health professional acted as a gatekeeper and mediated engagement and enrolment to ensure the right type of patients were registered for a telehealth service [49]. Altruistic reasons for engagement were also mentioned in one paper as some participants working at a university signed up for a DHI to support colleagues conducting research [41].

“*I make that decision by the patient’s need. If their diabetes is poorly controlled, then you need to use more tools to get them under control… you don’t really need it with all your patients with diabetes. You need it on the ones that need extra help.”* – Facilitator (CP-e) [49]

On the other hand, some people lacked an awareness of the existence of technology that could be used to support their health as it was not widely promoted. Public health education was not a fundamental aspect of some strategies used so people had a poor understanding of what a DHI could do, which meant they had little interest in signing up to use it [46, 47, 59]. A further problem was that some people had difficulties understanding the recruitment message, who it came from, why it was relevant to them or how to go about enrolling in a digital platform [41, 51]. A lack of clinical endorsement was a clear barrier for others who felt that if their healthcare provider would not promote digital health or use it themselves, then it was probably of limited value [46, 57]. On the other hand, if health professionals or trusted organisations affiliated with healthcare were supportive this reassured people it was worth signing up to [44, 52].

*“I would probably if I knew that the physician would access that prior to an appointment. If the physician didn’t read it, if it was more of a personal thing [just for me to do], I don’t know if I would kind of follow through with that.*” – Barrier (CP-i) [57]

#### Quality of the Digital Health Intervention (DHI)

The final theme affecting patients and the public’s ability to engage and enrol in digital health relates to the perceived quality of the information or interaction provided via technology. Some people signed up for a digital product or service as they felt it provided a more open communications channel with their healthcare provider [45, 57], while others gained the social support they needed online quickly and easily which enabled them to better manage their illness [52–54, 57]. In one paper, individuals cited medical errors they had personally experienced due to a lack of technology and welcomed electronic systems as a way to minimise mistakes and improve the quality of health data and care they received [47]. In addition, technology that was as automated as possible and integrated with other systems was seen to be more usable which would encourage enrolment [56].

*“I was so down and my peers/family couldn’t handle it and I needed someone who could tell me that it would be OK and that it was normal but also that I needed to stop feeling sorry for myself in a nice way…. I just went online and look for my support group [sic].”* – Facilitator (RM-s) [53]

However, others felt they would receive a poorer level of care due to the impersonal nature of electronic media as it lacked the nuances of human interaction, in particular where therapeutic relationships with clinicians were important social outlets or sensitive health issues were involved [45–48, 50, 54–57, 59].

*“I don’t think you would get the same feeling as if you were one-to-one in a room. You get more, you get to know the other person, so in a way you would. To me it would be like talking to a machine.”* – Barrier (RM-ia) [45]

In some cases the quality of health information accessed online was thought to be unreliable, without input from a qualified doctor or nurse, and the potential for identity fraud made it difficult for some people to trust advice from virtual health professionals [45, 55–57]. In one paper, abusive or threatening behaviour that could develop in virtual relationships was a barrier that prevented others from engaging and enrolling [50]. Finally, the usability of the DHI also featured under quality as some individuals felt they would not sign up if it was too slow and cumbersome to register or use it [41, 47, 48, 56].

### Developing a conceptual understanding of digital health engagement and recruitment processes

**Fig. 2:**
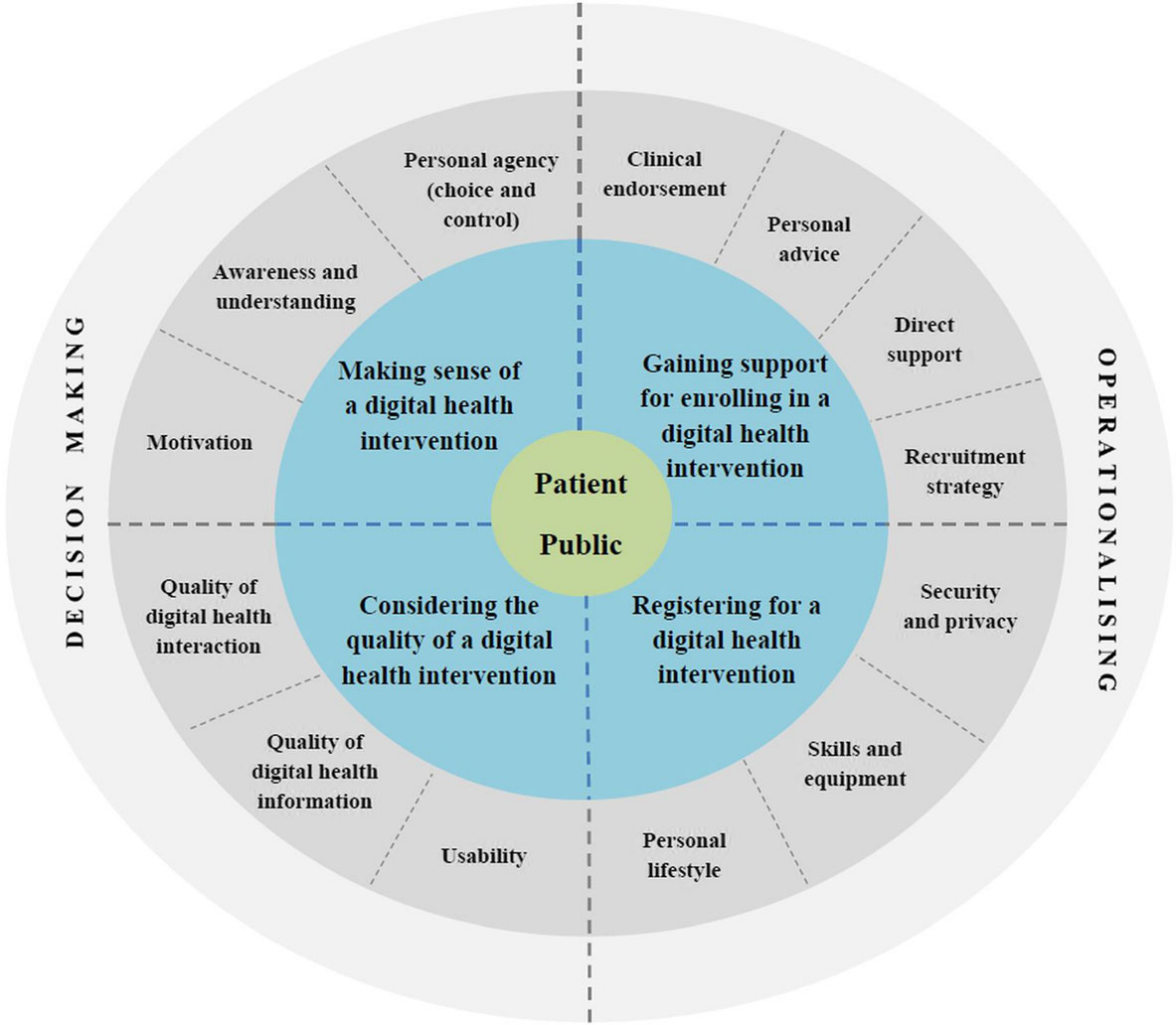
Digital Health Engagement Model (DIEGO)

We have used our catalogue of barriers and facilitators, conceptualised through the lens of NPT to develop an initial conceptual model of engagement and recruitment processes to help illuminate the myriad factors that affect patients’ and the publics’ ability to take part in digital health (see Fig. 2). This **DI**gital Health **E**n**G**agement M**O**del (DIEGO) centres on four main processes that people need to work through when first deciding if they wish to engage with a DHI (decision-making) and then when signing up to it (operationalising). In making a decision about whether to engage, people need to make sense of a DHI and consider its quality. From there, people must operationalise their decision by gaining adequate support to enrol and actively register for the DHI. Each of these four key processes are made up of a number of subcomponents that affect how patients and the public progress through the digital health engagement and enrolment journey. This preliminary conceptual model services as a useful heuristic to help people think through key engagement and enrolment issues that merit attention. Further investigation will be necessary to determine the relative importance of different elements of the model.

## Discussion

This review provides a summary of reported engagement and recruitment strategies, a catalogue of barriers and facilitators patients and the public experience when engaging and enrolling in DHIs as well as a preliminary conceptual model of key elements in this process. While none of the included papers comprehensively covered the entire process of engaging with and signing up to a DHI each study examined one or more aspects of people’s positive and negative experiences.

### Existing knowledge and future research

This systematic review explores how patients and the public engage with and enrol in a broad range of DHIs. Its findings support and expand those of an earlier review, which primarily looked at people accessing health information online [21]. One theme from that paper which affected engagement was the “characteristics of users”, such as their age, ethnicity, economic status and educational attainment; this did not emerge strongly from our review given the diversity of participants involved. However, the educational level people attain was one factor in our review that did affect engagement with digital health, as those with poor computer skills found it challenging to enrol which is in keeping with previous literature. In addition, as very few of the included studies in our review involved people over sixty years of age and other literature on usability points to older adults having more difficulties with digital health [60, 61], it would be wise to explore in more depth why this population do or do not engage with and enrol in DHIs. Similarly, ethnicity and socioeconomic status were not well described in the papers in this review so definitive conclusions about how culture and social position affects engagement with DHIs cannot be made. Literacy skills [62–64] and being able to pay for the technology [65] do impact on people’s ability to interact with and use DHIs, which is consistent with the findings of our review.

This review incorporated several different DHIs but newer platforms such as wearable devices are also emerging in this space [66] and more will undoubtedly follow as nanotechnology and biotechnology take off. It will therefore be important to update this review in due course to incorporate these new trends, expand on the taxonomy of engagement and enrolment strategies used to encourage people to sign up to them and the barriers and facilitators experienced in the process. However, it is likely that many of the same issues will emerge as the generative mechanisms of digital health engagement and enrolment have been teased out through our conceptual work when developing the new **DI**gital Health **E**n**G**agement M**O**del (DIEGO).

### Limitations

This review followed the ENTREQ guidelines for the reporting of systematic reviews of qualitative studies but it does have some limitations. The search strategy used introduced a number of constraints. Publications included were in the English language only; while this may have excluded potentially useful studies, there is evidence that limiting studies in this way does not introduce significant bias [67]. The search dates were limited to studies after the year 2000 but as this is a rapidly evolving sphere we believe this is justifiable. The selection criteria specifically excluded studies discussing recruitment to RCTs, as the focus here was on engagement and enrolment to “real-world” DHIs. Furthermore, many DHIs are developed in the commercial space and marketed to consumers but these have not been formally evaluated through rigorous research and so the literature is limited to only those applications that have undergone academic evaluation [68]. This does mean that some pertinent evidence could have been missed. The analysis and synthesis of the qualitative studies was based on our review of published data and not the original data, which may result in the loss of some important explanatory context. In addition, cultural differences in how people perceive and engage with DHIs, is not well understood, and the existing literature presents a predominantly Western viewpoint, which is a limitation. Furthermore, issues of socioeconomic disadvantage are not systematically addressed in the literature, which is another limitation. However, although more research will be required, based on the literature published to date, a number of recommendations are made about how to address the difficulties patients and the public face when engaging or enrolling in DHIs and what health professionals, health service managers, policy makers, industry and others need to consider to overcome these challenges.

#### Recommendation 1

This review has emphasised that people struggle to “make sense” of digital health and that it is not yet considered the “norm”. BOTT suggests that use of health services, which includes digital healthcare are social experiences that are “governed by expectations of accountability and norms of membership and behaviour” [38]. This leads us to suggest that:

*There is a need to invest in raising the profile of digital health products and services so patients and the public are knowledgeable about them.*

Work is needed to increase public awareness of different technologies and understanding of how they work, what benefits they can bring and potential risks inherent in using them. Further research is needed on novel ways to engage and educate the public about digital health as well as more investment in traditional forms of public health education [69]. Identifying which engagement and recruitment strategies are most effective for different groups of patients, consumers and technologies would also be beneficial [14, 70], as detailed descriptions of these were largely missing from the included studies. While communicating via mass media such as newspapers, television and radio advertising is becoming less popular as these services move online, the virtual space offers numerous opportunities to provide interactive educational content and promote collaborative sharing and learning, especially through social media [71]. However, this is dependent on patients and the public having access to digital platforms in the first instance, which as outlined in the review is not always feasible for some so more digital inclusion initiatives are necessary to address the digital divide [64]. Identifying and measuring which engagement and enrolment strategies are most effective for different groups of patients, consumers and technologies would also be beneficial to improve awareness and understanding of DHIs [14, 70], as detailed descriptions of these were largely missing from the included studies. A range of metrics could be developed, such as the cost of engaging an individual through a particular strategy or the time taken to recruit a critical mass of users via a certain method, to help determine which approaches are most successful and some such as web analytics are already in use [72]. It will be important that future studies describe engagement or recruitment strategies in greater detail to improve the fidelity and impact of these approaches [73]. Development of a template for engagement and enrolment strategies analogous to the one developed for intervention description and replication called the Template for Intervention Description and Replication (TIDieR) [74] would be helpful.

#### Recommendation 2

This review has shown that individuals consider several different quality aspects of a digital health product or service before signing up to it. The perceived value of the electronic health service interaction or the information people can convey and receive through digital means is a critical elements that must be better in one or more ways than the current standards of care to encourage people to register for it. BOTT reminds us that “relational networks can act as collective agents to negotiate and navigate healthcare services” [38]. Thus, we would suggest that:

*Technology that incorporates and enhances communication, social interaction and relationships with formal and informal care providers and peers with similar health issues, both online and offline, may help ensure engagement and enrolment, as people can quickly and easily access the social support they need to manage their wellbeing.*

Gamification [75], social networking applications [76] and wearable technologies [66] are currently being explored to improve the usability and social connectedness of digital health products and services and further work should explore how these can contribute to engagement and enrolment. There is growing evidence that additional support, such as peer support, can be an effective strategy for reaching individuals that healthcare has traditionally described as “hard to reach” [77, 78]. More research examining whether or how these new platforms can help address the different barriers to engagement and recruitment would be useful.

#### Recommendations 3 and 4

This review has emphasised that gaining the right support to enrol is another important element in digital health engagement and recruitment processes. This support can take numerous forms but it is clear that clinical endorsement from trusted health professionals or organisations is helpful in getting people to engage and sign up to digital health. For consumer facing technology personal recommendations and direct help from family and friends can be useful. Drawing on BOTT’s relational networks [38] to direct engagement with digital health we suggest that:

*Accreditation and endorsement by respected clinical organisations or clinicians will be an important factor promoting engagement with digital health.*

And

*Marketing and engagement activities should consider targeting not just the individuals with a given condition or health issue but their wider relational and support networks, whose input may be a crucial factor in deciding uptake of new digital health initiatives.*

More research on whether DHIs should be accredited and approved by healthcare organisations and clinicians and how this should be done, given legal and ethical implications, would also be useful to provide guidance to individual healthcare professionals as well as local and national health services on how to promote engagement in digital health [68]. Health professionals have been known to act as gatekeepers to DHIs and block patient recruitment [11]. More research on how to address this issue would be beneficial as it is an important avenue by which patients and the public can learn about DHIs and enrol in them.

#### Recommendation 5

The published literature to date is clear that even if a DHI is high quality, well publicised and promoted, and patients and consumers are aware of and supported to sign up to it, there is no guarantee that they will register for it as other factors can affect their ability to enrol. In particular, busy lifestyles, with competing demands on individuals for their time and commitment, often taken precedence over personal health. As BOTT highlights people’s “functional performance” is mediated by their cognitive and material capacity and “exercise of agency is constrained by controls on service content and the distribution of opportunities of care, and by the social and economic resources available” [38]. This leads us to suggest that:

*Digital health engagement and enrolment strategies along with the products and services should be better designed and tailored where possible to lessen rather than increase the self-care burden of treatment people endure. This could enable them to integrate digital health with their current lifestyle, as a one-size fits all approach is unlikely to be effective.*

As disease trends change over time DHIs must be designed in a flexible manner to accommodate the changing demographic and health landscape. For example, as multimorbidity becomes more commonplace it will impact on the future design requirements of many DHIs, which typically have a single disease focus and are not yet capable of providing holistic self-management solutions for patients and the public [79]. In the future, DHIs may also need to combine the health and social care needs of individuals, as these are often closely intertwined, and some health systems are now moving towards integrating health and social care services [80, 81]. Research in this space is exploring personalising technology through co-design and other participatory methods to improve usability as patients and the public are often excluded from this process and their input will be vital if DHIs are to be successful [82, 83]. Furthermore, digital health readiness assessments are under development to see if an individual has the capacity for a DHI, what form this should take, and what engagement and enrolment strategies suit them [84]. More work in this area would be beneficial and DIEGO could be a starting point for the development of future digital health readiness toolkits that focus on the patient and consumer perspective.

The review has reinforced the fact that usability is a significant factor in a person’s decision to sign up to a DHI. Therefore, digital platforms should have simple and short enrolment processes and it is essential that the systems themselves are easy to use so they are not burdensome, as this is a key factor that will affect uptake. In addition, people expect more integrated and automated systems that are continuously available. Interoperability issues between technologies and electronic systems are currently being tackled [85] and the development of application programming interfaces [86] are helping to close this gap further but more work on how to provide seamless digital health services would be helpful to encourage patients and the public to sign up to them.

#### Recommendation 6 and 7

This review has also highlighted that poor digital literacy skills, the cost of some technologies and the fact that high speed Internet access is still not ubiquitous prevents many people from signing up for a digital health intervention. In keeping with the exercise of agency expressed in BOTT [38] we suggest that:

*More investment in digital upskilling mechanisms and technical infrastructure is needed alongside engagement and recruitment strategies if digital health uptake is to be enhanced.*

And

*Better funding models need to be put in place to help ensure equity of access to digital health products and services.*

Research in this space is emerging [63–65] but further work is necessary to illuminate the best means of achieving this for different groups of patients and the public. There is an assumption that these issues will become less of a factor over time, as the younger generation who are more digitally literate get older, and 4G and 5G telecommunication networks are rolled out. However, there is evidence that the penetration of technology in society does not guarantee that adolescents have more chance to learn and use IT, as numerous factors such as home IT access, gender and socio-economic status can affect children’s digital skills [87] and recent statistics show older adults still continue to struggle to use digital health [88]. While many countries are investing in upgrading their network capacity, the ability to pay for technology whether it is the hardware, software, network connectivity or data consumption necessary to utilise DHIs will not always be feasible for some people [89], especially those in low and middle-income nations. Therefore, to prevent further inequalities in health developing more work on these issues is necessary.

#### Recommendation 8

Finally, this review underscores that security and privacy of personal information and the anonymity of digital health platforms affect engagement. Whether patients and the public consider their data to be safe, secure and used appropriately by those who control and manage it is a consideration they make before enrolling. BOTT underscores the importance of “social capital”, which is access to information and material resources, to enhance people’s “structural resilience” or the ability to adapt to adversity and treatment burden [38]. Thus, we suggest that:

*The public should be made more aware of the potential security risks with digital health products and services and better regulations need to be put in place to protect them to encourage engagement.*

Given that some technology sectors such as the mobile app industry are completely unregulated [90–92] and cybercrime is prevalent [93], it would also be pertinent to inform the public about the potential risks involved in using digital health products and services and what is being done to protect the privacy and security of their data.

## Conclusion

It is clear from our framework of barriers and facilitators that digital health engagement and recruitment processes are complex, with many interconnecting factors that affect patients’ and the public’s ability to engage and enrol in a technology and there remains outstanding gaps in knowledge. Our preliminary Digital Health Engagement Model (DIEGO) provides a useful checklist for health professionals, health service managers, policy makers, academia, industry and others to consider when implementing digital health in the real world and will be particularly helpful for newcomers to the field. Future research must aim to describe engagement or enrolment strategies in greater detail, including theoretical underpinnings if we are to more effectively study, classify, and learn which approaches are more likely to succeed.

## Additional files

Additional file 1: PubMed Search Strategy. (DOCX 16 kb) Additional file 2: Data Extraction Template. (DOCX 15 kb) Additional file 3: Details of Studies and Participants. (XLSX 23 kb) Additional file 4: COREQ Checklist. (XLSX 61 kb) Additional file 5: Participant Quotes. (DOCX 25 kb)

## Abbreviations

BOTT: Burden of treatment theory
COREQ: Consolidated criteria for reporting qualitative research
DIEGO: Digital health engagement model
ENTREQ: Enhancing transparency in reporting the synthesis of qualitative research
IVR: Interactive voice recognition
NPT: Normalization process theory
PCs: Personal computers
PHRs: Personal health records
RCT: Randomized controlled trials
SMS: Short message service
TIDieR: Template for intervention description and replication
