# Supplementary material for: Understanding factors affecting patient and public engagement and recruitment to digital health interventions: a systematic review of qualitative studies
Source: BMC Med Inform Decis Mak. 2016 Sep 15;16:120. doi: 10.1186/s12911-016-0359-3 (PMC5024516; doi:10.1186/s12911-016-0359-3)
Supplement: Additional file 1: — PubMed Search Strategy. (DOCX 16 kb) [file 12911_2016_359_MOESM1_ESM.docx]

**Additional file 1: PubMed Search Strategy.**

Interface/URL: <http://www.ncbi.nlm.nih.gov/pubmed>

Date of search: 19/08/2015

Search Strategy:

#01 Search Online Systems[mh:noexp] 7190

#02 Search Medical Informatics[mh:noexp] 8373

#03 Search Medical Informatics Applications[mh:noexp] 2059

#04 Search Educational Technology[mh:noexp] 1130

#05 Search Electronics, Medical[mh:noexp] 6164

#06 Search Audiovisual Aids[mh:noexp] 6192

#07 Search Telecommunications[mh:noexp] 4341

#08 Search Multimedia[mh:noexp] 1505

#09 Search Hypermedia[mh:noexp] 388

#10 Search Cell Phones[mh:noexp] 4763

#11 Search Social Networking[mh:noexp] 928

#12 Search Telemedicine[mh:noexp] 11652

#13 Search Telenursing[mh:noexp] 126

#14 Search Telephone[mh:noexp] 9247

#15 Search Ambulatory Care Information Systems[mh:noexp] 1157

#16 Search Mobile Applications[mh:noexp] 255

#17 Search Wireless Technology[mh:noexp] 1161

#18 Search Electronic Mail[mh:noexp] 1890

#19 Search Electronic Health Records[mh:noexp] 6972

#20 Search (("personal health record" [tiab] OR "personal electronic health record" [tiab] OR PHR [tiab]) 1047

#21 Search (phone*[tiab] OR mobile*[tiab] OR smartphone*[tiab] OR handset*[tiab] OR hand-set*[tiab] OR handheld*[tiab] OR hand-held*[tiab]) 87377

#22 Search ((electronic*[tiab] OR digital*[tiab] OR device*[tiab]) AND tablet*[tiab]) 1344

#23 Search ("tablet PC"[tiab] OR "tablet computer"[tiab]) 223

#24 Search device-based[tiab] 1398

#25 Search ((digital*[tiab] OR electronic*[tiab] OR communicat*[tiab]) AND device*[tiab]) 22166

#26 Search ((device*[tiab] AND technolog*[tiab])) 19965

#27 Search ((PDA[tiab] OR PDAs[tiab] OR "personal digital"[tiab])) 6978

#28 Search (mp3-player*[tiab] OR mp4-player*[tiab]) 89

#29 Search (online[tiab] OR on-line[tiab] OR internet[tiab] OR www[tiab] OR web[tiab] OR website*[tiab] OR webpage*[tiab] OR broadband[tiab] OR broad-band[tiab]) 174772

#30 Search (wireless[tiab] OR wire-less[tiab] OR wifi[tiab] OR wi-fi[tiab] OR "global positioning system*"[tiab] OR bluetooth*[tiab]) 7972

#31 Search (text messag*[tiab] OR texting[tiab] OR texter*[tiab] OR texted[tiab] OR SMS[tiab] OR short messag*[tiab] OR multimedia messag*[tiab] OR multi-media messag*[tiab] OR mms[tiab] OR instant messag*[tiab]) 8062

#32 Search (social media*[tiab] OR facebook[tiab] OR twitter[tiab] OR tweet[tiab] OR tweets[tiab]) 2766

#33 Search (webcast*[tiab] OR webinar*[tiab] OR podcast*[tiab] OR wiki[tiab] OR wikis[tiab] OR youtube[tiab] OR you tube[tiab] OR vimeo[tiab]) 1452

#34 Search (app[tiab] OR apps[tiab]) 14179

#35 Search ((electronic*[tiab] OR digital*[tiab] OR device*[tiab]) AND application*[tiab]) 53728

#36 Search (iphone*[tiab] OR i-phone*[tiab] OR ipad*[tiab] OR i-pad*[tiab] OR ipod*[tiab] OR i-pod*[tiab] OR palm os[tiab] OR "palm pre classic*"[tiab]) 1160

#37 Search (android*[tiab] OR ios[tiab] OR s40[tiab] OR symbian*[tiab] OR windows[tiab]) 14731

#38 Search (video*[tiab] OR dvd[tiab] OR dvds[tiab]) 66751

#39 Search (email*[tiab] OR e-mail*[tiab] OR electronic mail*[tiab]) 9154

#40 Search (chat room*[tiab] OR chatroom*[tiab]) 268

#41 Search (blog*[tiab] OR blogging[tiab] OR blogger*[tiab] OR weblog*[tiab]) 888

#42 Search skype[tiab] 112

#43 Search (bulletin board*[tiab] OR bulletinboard*[tiab] OR messageboard*[tiab] OR message board*[tiab]) 421

#44 Search (software*[tiab] OR soft-ware*[tiab]) 93613

#45 Search (interactiv*[tiab] OR inter-activ*[tiab]) 35876

#46 Search (ehealth*[tiab] OR e-health*[tiab] OR mhealth*[tiab] OR m-health*[tiab] OR m-learning[tiab]) 2596

#47 Search (electronic learn*[tiab] OR e-learn*[tiab]) 1367

#48 Search (telephone*[tiab] OR telehealth[tiab] OR telemedicine[tiab] OR telenursing[tiab] OR telemonitor*[tiab]) 50718

#49 Search ((digital*[tiab] OR electronic*[tiab] OR communicat*[tiab] OR information*[tiab]) AND technolog*[tiab]) 55799

#50 Search ((digital*[tiab] OR electronic*[tiab]) AND (intervention*[tiab] OR therap*[tiab] OR treatment*[tiab] OR medicine[tiab] OR medical*[tiab] OR health*[tiab])) 78019

#51 Search (ICT[tiab] OR ICTs[tiab]) 3070

#52 Search medical informatics[tiab] 1782

#53 Search (remot*[tiab] AND (care[tiab] OR caring[tiab] OR cared[tiab] OR manag*[tiab] OR consult*[tiab] OR monitor*[tiab] OR measur*[tiab])) 18099

#54 Search (#1 OR #2 OR #3 OR #4 OR #5 OR #6 OR #7 OR #8 OR #9 OR #10 OR #11 OR #12 OR #13 OR #14 OR #15 OR #16 OR #17 OR #18 OR #19 OR #20 OR #21 OR #22 OR #23 OR #24 OR #25 OR #26 OR #27 OR #28 OR #29 OR #30 OR #31 OR #32 OR #33 OR #34 OR #35 OR #36 OR #37 OR #38 OR #39 OR #40 OR #41 OR #42 OR #43 OR #44 OR #45 OR #46 OR #47 R #48 OR #49 OR #50 OR #51 OR #52 OR #53) 145894

#55 Search (recruitment strateg*[tiab] OR recruitment method*[tiab]) 1657

#56 Search (recruit*[tiab] AND (patient[tiab] OR patients[tiab] OR volunteer*[tiab] OR participant*[tiab] OR people[tiab] OR person*[tiab] OR woman[tiab] OR women[tiab] OR man[tiab] OR men[tiab] OR child[tiab] OR children[tiab] OR elder[tiab] OR elderly[tiab] OR students[tiab] OR adolescen*[tiab] OR rural[tiab])) 127518

#57 Search ((participation[tiab] OR participating[tiab]) AND (patient[tiab] OR patients[tiab] OR volunteer*[tiab] OR participant*[tiab] OR people[tiab] OR person*[tiab] OR woman[tiab] OR women[tiab] OR man[tiab] OR men[tiab] OR child[tiab] OR children[tiab] OR elder[tiab] OR elderly[tiab] OR students[tiab] OR adolescen*[tiab] OR rural[tiab])) 91719

#58 Search ((sign up[tiab] OR take up[tiab] OR enlist[tiab]) AND (patient[tiab] OR patients[tiab] OR volunteer*[tiab] OR participant*[tiab] OR people[tiab] OR person*[tiab] OR woman[tiab] OR women[tiab] OR man[tiab] OR men[tiab] OR child[tiab] OR children[tiab] OR elder[tiab] OR elderly[tiab] OR students[tiab] OR adolescen*[tiab] OR rural[tiab])) 1947

#59 Search ((engagement[tiab] OR engage[tiab] OR engaging[tiab) AND (patient[tiab] OR patients[tiab] OR volunteer*[tiab] OR participant*[tiab] OR people[tiab] OR person*[tiab] OR woman[tiab] OR women[tiab] OR man[tiab] OR men[tiab] OR child[tiab] OR children[tiab] OR elder[tiab] OR elderly[tiab] OR students[tiab] OR adolescen*[tiab] OR rural[tiab])) 37503

#60 Search ((involvement[tiab] OR involve[tiab] OR involving[tiab]) AND (patient[tiab] OR patients[tiab] OR volunteer*[tiab] OR participant*[tiab] OR people[tiab] OR person*[tiab] OR woman[tiab] OR women[tiab] OR man[tiab] OR men[tiab] OR child[tiab] OR children[tiab] OR elder[tiab] OR elderly[tiab] OR students[tiab] OR adolescen*[tiab] OR rural[tiab])) 320397

#61 Search ((enrolment[tiab] OR enrollment[tiab] OR enrol[tiab] OR enroll[tiab] OR enrolling[tiab] OR enrolled[tiab) AND (patient[tiab] OR patients[tiab] OR volunteer*[tiab] OR participant*[tiab] OR people[tiab] OR person*[tiab] OR woman[tiab] OR women[tiab] OR man[tiab] OR men[tiab] OR child[tiab] OR children[tiab] OR elder[tiab] OR elderly[tiab] OR students[tiab] OR adolescen*[tiab] OR rural[tiab])) 173815

#62 Search (invit*[tiab] AND (patient[tiab] OR patients[tiab] OR volunteer*[tiab] OR participant*[tiab] OR people[tiab] OR person*[tiab] OR woman[tiab] OR women[tiab] OR man[tiab] OR men[tiab] OR child[tiab] OR children[tiab] OR elder[tiab] OR elderly[tiab] OR students[tiab] OR adolescen*[tiab] OR rural[tiab])) 19828

#63 Search Consumer Behavior[mh:noexp] 17705

#64 Search Consumer Participation[mh:noexp] 14268

#65 Search Patient Participation[mh:noexp] 18279

#66 Search Social Participation[mh:noexp] 669

#67 Search Community-Based Participatory Research[mh:noexp] 2105

#68 Search ((difficult*[tiab] OR problem*[tiab] OR deterrent*[tiab] OR obstacle*[tiab] OR hindrance*[tiab] OR barrier*[tiab] OR challenge*[tiab] OR impediment*[tiab] OR experience*[tiab]) AND (access[tiab] OR participation[tiab] OR engagement[tiab] OR enrollment[tiab] OR enrolment[tiab] OR recruitment[tiab] OR uptake[tiab])) 128946

#69 Search Communication Barriers[mh:noexp] 4855

#70 Search (#55 OR #56 OR #57 OR #58 OR #59 OR #60 OR #61 OR #62 OR #63 OR #64 OR #65 OR #66 OR #67 OR #68 OR #69) 850208

#71 Search (#54 AND #70) 18218

#72 Search (animals[mh] not humans[mh:noexp]) 3975150

#73 Search ((editorial[pt] OR news[pt] OR case reports[pt]) NOT randomized controlled trial[pt]) 2241721

#74 Search case report[ti] 168264

#75 Search (#72 OR #73 OR #74) 6205772

#76 Search (#71 NOT #75) 17694

Applied date limit from 2000/01/01 to 2015/08/19
