# Supplementary material for: Understanding factors affecting patient and public engagement and recruitment to digital health interventions: a systematic review of qualitative studies
Source: BMC Med Inform Decis Mak. 2016 Sep 15;16:120. doi: 10.1186/s12911-016-0359-3 (PMC5024516; doi:10.1186/s12911-016-0359-3)
Supplement: Additional file 2: — Data Extraction Template. (DOCX 15 kb) [file 12911_2016_359_MOESM2_ESM.docx]

**Additional file 2: Data Extraction Template.**

| **ARTICLE DETAILS** | | | | |
| --- | --- | --- | --- | --- |
| Study Title |  | | | |
| Authors |  | | | |
| Journal, Vol, Issue, Page(s) |  | | | |
| Year |  | | | |
| DOI/Article ID |  | | | |
| **Digital Health Intervention (DHI)** |  | | | |
| Telehealth system/application |  | | | |
| Mobile application or SMS service |  | | | |
| Online or web-based service |  | | | |
| Other |  | | | |
| Unclear |  | | | |
| **Engagement/Recruitment strategy** |  | | | |
| Health or social care professional |  | | | |
| Traditional mass marketing e.g. TV, radio, newspaper advertisement |  | | | |
| Internet and Social Media |  | | | |
| Other |  | | | |
| Unclear |  | | | |
| **Engagement/Recruitment process** |  | | | |
| What did the engagement or recruitment process consist of? |  | | | |
| **Setting of DHI** |  | | | |
| Home |  | | | |
| Workplace |  | | | |
| Community e.g. family practice, nursing or care home, rehabilitation centre |  | | | |
| Hospital inpatient |  | | | |
| Outpatient clinic |  | | | |
| Other |  | | | |
| Unclear |  | | | |
| **Study Details** | **Provided** | **Not Provided** | | **Unclear** |
| What is the research question or research aim(s)? |  |  | |  |
| What sampling procedure is used to select participants? |  |  | |  |
| What form of data collected is used? |  |  | |  |
| What form of data analysis is used? |  |  | |  |
| What is the overall conclusion or recommendations of the study? |  |  | |  |
| What (if any) study limitations are declared? |  |  | |  |
| How is the study funded? Are any conflicts of interest declared? |  |  | |  |
| **Participant Details** |  | | | |
| Inclusion criteria |  | | | |
| Exclusion criteria |  | | | |
| Number of Participants |  | | | |
| Types of Participants |  | | | |
| Min age of participants |  | | | |
| Max age of participants |  | | | |
| Number of males |  | | | |
| Number of females |  | | | |
| Chronic or other health condition |  | | | |
| Socioeconomic status |  | | | |
| Ethnicity |  | | | |
| **Quote** | **Barrier / Facilitator** | | **NPT Code** | |
|  |  | |  | |
|  |  | |  | |
