# Supplementary material for: Understanding factors affecting patient and public engagement and recruitment to digital health interventions: a systematic review of qualitative studies
Source: BMC Med Inform Decis Mak. 2016 Sep 15;16:120. doi: 10.1186/s12911-016-0359-3 (PMC5024516; doi:10.1186/s12911-016-0359-3)
Supplement: Additional file 5: — Participant Quotes. (DOCX 25 kb) [file 12911_2016_359_MOESM5_ESM.docx]

**Additional file 5: Participant quotes for each major theme and subtheme coded to Normalization Process Theory.**

| **Theme 1: Personal Agency and Motivation** |
| --- |
| **Subtheme 1.1: Motivation** |
| **Quote 1:** "*[I subscribed] to get the reminders, because if you're sat, if you are in a lunch break and you're sat at your desk just on the Internet and you're not moving and you're eating something that's not good and then you get a reminder and it's just: 'have a walk!', or something. Straight away there is a trigger in your mind and you think: 'yeah, that's right, I can do that!" –* Facilitator (CO-i) [41]  **Quote 2:** *“For me, it does not change anything because I am always in a car. I walk very little so I will feel even guilty for not having walked. I will look down at the low numbers and I’ll feel anxious.” –* Barrier (CO-is) [42] |
| **Subtheme 1.2: Awareness and understanding** |
| **Quote 1:** “*Anything you can learn is helpful. When you have something, you want to know everything about it, the good and the bad. What can happen to you if you don’t eat properly or medicines don’t take? I want to know the worst and the best*.” – Facilitator (CO-is) [59]  **Quote 2:** *“Many were unsure of the purpose of HealthSpace, describing it as “pointless,” “irrelevant,” and not fit for purpose (“I would just rather write it down in the diary or just hide it underneath my bed or something”)”* – Barrier (CO-i) [55] |
| **Subtheme 1.3: Personal agency (choice and control)** |
| **Quote 1:** *“One thing that appeals to me is that you could do it immediately, as opposed to having to book an appointment with a clinician and maybe you won’t be able to do that for a few days. Especially if I was very concerned about something and wanted answers immediately.”* – Facilitator (Co-d) [50]  **Quote 2:** *“I just decided it wasn't worth my while because I cycle fifteen miles a day, so you know, I probably couldn't do much more exercise anyway. I've got my own exercise routine.” -* Barrier (CO-d) [41] |
| **Theme 2: Personal Life and Values** |
| **Sub-theme 2.1: Personal lifestyle** |
| **Quote 1:** *"This is definitely a service I would use, not only for the convenience factor but I mean, no matter how old we are, it’s still an embarrassing issue for a lot of people.”* – Facilitator (CA-iw) [46]  **Quote 2: “***I didn’t sign up or I didn't do the programme for any other reason than simply due to constraints on my time and difficulties on my time, otherwise I think I would have gladly welcomed the participation. I work full time, and I've issues with my personal life, so I didn't really have a huge amount of time to do any sort of things****”* –** Barrier (CA-ci) [41] |
| **Sub-theme 2.2: Skills and equipment** |
| **Quote 1:** *"I presume that like technology is maybe the right way forward with this. Because that’s, you never see a young person that does not have a mobile phone."* – Facilitator (CA-sw) [48]  **Quote 2:** "*I’m not tech savvy, so, I’m from the “old school” and I hate the cell phones my children give me."* – Barrier (CA-sw) [57] |
| **Sub-theme 2.3: Security and privacy** |
| **Quote 1: *“****While not a single participant thought that these measures would guarantee the security of their data, most thought that the small risk of identity fraud, disclosure, or blackmail was worth taking. They contrasted personal health information (seen as a low security risk) with their bank details (much higher risk), and some people with serious illness joked that nobody would want to steal their identity****”*** – Facilitator (CA-ri) [55]  **Quote 2:** *“I’m very wary of the internet, we leave digital footprints wherever we go and you never know what’s going to come back and haunt you and I think the more that you are in a professional working environment the more you need to be careful about what you put online. You’ve got to keep it within certain parameters.”* – Barrier (CA-ri) [49] |
| **Theme 3: Engagement and Recruitment Approach** |
| **Subtheme 3.1: Recruitment strategy** |
| **Quote 1:** “*I make that decision by the patient's need. If their diabetes is poorly controlled, then you need to use more tools to get them under control... you don't really need it with all your patients with diabetes. You need it on the ones that need extra help.”* – Facilitator (CP-e) [44]  **Quote 2:** *“some parents did not enroll because they were apprehensive about signing up for an SMS program. These parents, who saw recruitment materials but did not speak with program staff, reported worrying about how much the program would cost them, how long they would have to remain enrolled, and the exact content of the messages.” –* Barrier (CP-l) [51] |
| **Subtheme 3.2: Direct support** |
| **Quote 1:** *“Two carers said that the patient did not have the skills to register or use the technology themselves, and another participant (visually impaired) needed a partner’s help because the grid card was not available in large print”* – Facilitator (CP-a) [56]  **Quote 2:** *"I was encouraged to sign up by my old boss at that time, he didn't really tell us about that thing. He encouraged just to sign up so I did and then, once I had, I didn't really hear anything else about it and I didn't know what it was all to be honest, really what it was about or anything."* – Barrier (CP-e) [41] |
| **Subtheme 3.3: Personal advice** |
| **Quote 1:** *“It was a friend that recommended it last time we see: she had seen the posters and recommended it to me, because she knew I might have been interested.”* – Facilitator (CP-e) [41]  **Quote 2:** *“I just thought that our husbands or mates- not that they don’t want us to be healthy and learn about this - but they also are feeling time constraints. Maybe if they had an information session at the beginning to underline how important this is… what it’s going to entail, that they might have to give up a little bit of their time for us to do that*.” – Barrier (CP-a) [42] |
| **Subtheme 3.4: Clinical endorsement** |
| **Quote 1:** *“If it was accredited by a university or medical college or something like that it would be a good start.”* – Facilitator (CP-l) [49]  **Quote 2:** *"I would probably if I knew that the physician would access that prior to an appointment. If the physician didn’t read it, if it was more of a personal thing [just for me to do], I don’t know if I would kind of follow through with that.*" – Barrier (CP-i) [59] |
| **Theme 4: Quality of the Digital Health Intervention** |
| **Sub-theme 4.1: Quality of digital health information** |
| **Quote 1:** *“I will feel more comfortable to join the Chinese cancer support group, due to the language the same, and especially the culture the same. The jokes we make will be understandable, a lot of time we care about what is happening back to our origin country.”* – Facilitator (RM-ia) [58]  **Quote 2:** *"I assume that my doctor will inform me regardless [not] just because I have access to this that I am going to be on it."* – Barrier (RM-ia) [59] |
| **Sub-theme 4.2: Quality of digital health interaction** |
| **Quote 1:***“I was so down and my peers/family couldn’t handle it and I needed someone who could tell me that it would be OK and that it was normal but also that I needed to stop feeling sorry for myself in a nice way…. I just went online and look for my support group [sic].”* – Facilitator (RM-s) [58]  **Quote 2:** *"I don't think you would get the same feeling as if you were one-to-one in a room. You get more, you get to know the other person, so in a way you would. To me it would be like talking to a machine."* – Barrier (RM-ia) [53] |
| **Sub-theme 4.3: Usability** |
| **Quote 1:** “*It would be nice if you didn’t need to print anything out. If you could just e-mail it to the lab, and … then just kind of show up*.” – Facilitator (RM-r) [50]  **Quote 2:** “*I think the conception with e-mail is that you’re gonna have to wait a couple days for an answer. And, when you’re looking for an answer that can seem like a year*.” – Barrier (RM-ia) [50] |
